# Promise and Challenges of Checkpoint Inhibitor Therapy for Progressive Multifocal Leukoencephalopathy in HIV

**DOI:** 10.1007/s11904-022-00626-w

**Published:** 2022-10-01

**Authors:** Sydney Corey, Bryan R. Smith, Irene C. M. Cortese

**Affiliations:** 1 Experimental Immunotherapeutics Unit, National Institute of Neurological Disorders and Stroke, National Institutes of Health, 10 Center Drive, 5C103, Bethesda, MD 20892-1684 USA; 2 Section of Infections of the Nervous System, National Institute of Neurological Disorders and Stroke, National Institutes of Health, Bethesda, MD USA

**Keywords:** Progressive multifocal leukoencephalopathy, JCV, HIV, Checkpoint inhibitors, PD-1, Immune reconstitution inflammatory syndrome

## Abstract

**Purpose of Review:**

Progressive multifocal leukoencephalopathy (PML) is a severe opportunistic infection that remains an important cause of morbidity and mortality in people living with HIV (PLWH). Immune checkpoint molecules are negative regulators of the immune response that have been targeted as a strategy to bolster anti-viral immunity in PML, with varied outcomes reported. While initiation and optimization of antiretroviral therapy remains the standard of care in HIV-related PML, the specific opportunities and risks for checkpoint blockade in these cases should be explored.

**Recent Findings:**

As of April 15, 2022, only 5 of the 53 total published cases of PML treated with checkpoint blockade had underlying HIV infection; four of these had a favorable outcome. The risk of promoting immune reconstitution inflammatory syndrome is a major concern and underscores the importance of patient selection and monitoring.

**Summary:**

Checkpoint blockade warrants further exploration as a potentially promising option for treatment escalation in HIV-related PML.

**Supplementary Information:**

The online version contains supplementary material available at 10.1007/s11904-022-00626-w.

## Introduction

Progressive multifocal leukoencephalopathy (PML) is a rare and often fatal central nervous system (CNS) disease caused by the JC virus (JCV) [1]. The transmissible form of JCV, or archetype variant, commonly infects the general population in childhood, establishing asymptomatic latent or persistent infection. In individuals with compromised cellular immunity, however, JCV can reactivate and undergo serial genomic mutations; this in turn can result in the acquired ability of the virus to infect CNS glial cells with development of PML [2]. Even in those with immune deficiencies, the rarity of these sequential genomic rearrangements may in part explain the low estimated incidence of PML of 1:200,000 [3, 4].

The major underlying conditions contributing to PML cases include human immunodeficiency virus (HIV) infection, hematological malignancy, and exposure to immunomodulatory therapies [5]. In contrast with most opportunistic infections, no antiviral treatment currently exists for PML. As such, the only viable treatment strategies are aimed at rapidly achieving immune reconstitution. These include standard-of-care introduction of antiretroviral therapy (ART) in HIV-related PML, and discontinuation of any immunosuppressive treatments. However, in many patients with PML, reversing the underlying immunosuppression may not be accomplished, which has led to experimental treatment strategies aimed at augmenting antiviral immune responses [1].

Advancing these therapies is challenging due to the rarity and severity of PML, presenting ethical and methodological concerns regarding clinical trial design. Moreover, no animal model exists for PML and the virus is difficult to propagate in culture [1]. The collective treatment experience for PML is thus largely limited to case reports or small case series, making it difficult for the treating physician to weigh treatment options beyond standard-of-care.

## PML in HIV

Despite significant improvements in the management of HIV infection and associated complications, PML remains an important cause of morbidity and mortality among people living with HIV (PLWH). Prior to the AIDS pandemic, PML was first described as a rare occurrence in lymphoproliferative disease [6]. The AIDS pandemic resulted in a dramatic 12-fold increase in the frequency of PML between the early 1980s and early 1990s due to the significant immunosuppression of HIV infection [7]. The introduction of ART for PLWH has since lessened the impact of PML on this population. The incidence of PML decreased from a peak estimate of 14.8 cases per 1000 patient-years at risk (PYR) pre-ART to 2.6 cases per 1000 PYR during early years of ART in 2005, with a further decline to 0.8 cases per 1000 PYR by 2011 [8].

While widespread availability of ART and a greater recognition of both PML and PML-IRIS have improved survival from a dismal 10% in the pre-ART era [7], survival remains poor at approximately 50% [9, 10, 11••], underscoring the continued devastating consequences of JCV infection. Indeed, even among survivors, the majority are left with significant disability, explained by the extensive tissue injury associated with PML with marked volume loss observed on brain MRIs [9, 12••].

Although the past decade has seen a decline in the proportion of patients with HIV-related PML, cohort studies show HIV remains the single most frequent underlying condition, accounting for approximately 50% of total PML cases [12••, 13, 14••]. The distinct predisposition to PML in the setting of HIV infection remains incompletely unexplained. Synergistic effects of PML and HIV coinfection have been proposed. One hypothesis suggests HIV infection affects the CNS microenvironment with an up-regulation of adhesion molecules on endothelial cells which may facilitate the trafficking of JCV-infected B cells into the brain [13]. Moreover, the HIV trans-regulatory protein Tat has been reported to increase the activity of the JCV late promoter [15] and to enhance replication of JCV [1, 16]. Exploring this association further may provide insight when assessing what types of therapies may prove most beneficial for HIV-related PML. The continued impact of PML in PLWH, with persistently high morbidity and mortality rates, warrants the exploration of adjunctive therapies for HIV-associated PML.

## Immune Checkpoint Inhibitors

**Fig. 1:**
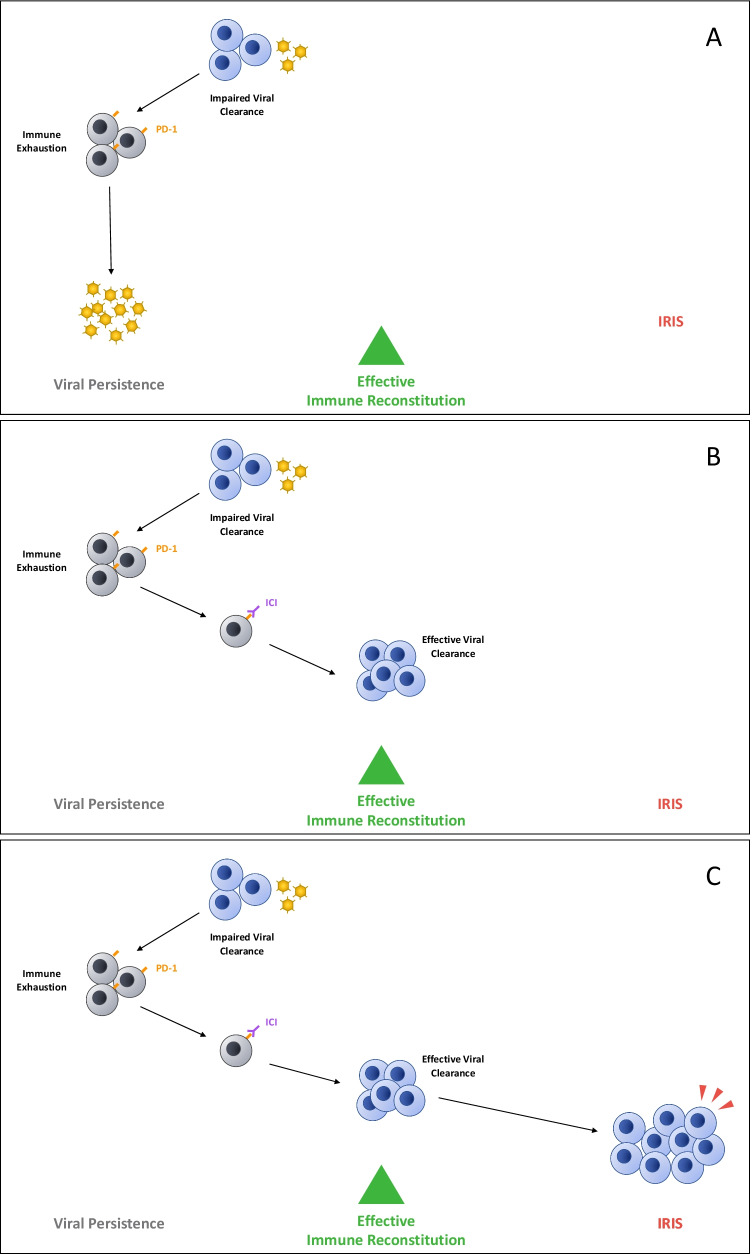
In the setting of impaired viral clearance and chronic exposure to high antigen burden, immune exhaustion may progressively dampen anti-viral immune responses leading to viral persistence (**A**). Treatment with immune checkpoint inhibitors (ICI), such as PD-1 inhibitors, may restore anti-viral immune responses and lead to effective viral clearance (**B**). The tipping point between effective immune reconstitution and immune activation inflammatory syndrome (IRIS) (**C**) is marked by development of new or worsening clinical symptoms attributable to the immune response itself rather than progression of the infection. In the setting of ART, adjunctive treatment with ICI could promote this overshoot

Immune checkpoint inhibitors (ICIs) have revolutionized the therapeutic approach to cancer treatment, opening the door for their application to re-establish immune competence in other diseases. ICIs block the inhibitory regulatory immune checkpoint pathways underlying immune exhaustion, thereby rescuing effective immune responses [17] (Fig. 1). The Food and Drug Administration (FDA) approved ipilimumab as the first ICI for the treatment of unresectable or metastatic melanoma in 2011 [18]. ICIs have since been widely applied to the treatment of a variety of cancers once thought less susceptible to treatment [17, 19]. Despite this success, the majority of patients with cancer do not respond to checkpoint inhibitors and so the identification of predictive biomarkers is an active area of investigation [20, 21].

The mechanism of action of ICIs suggests their potential for restoring immune function in other diseases. T cell exhaustion is a feature of numerous cancers and chronic infections, such as PML [4, 22, 23]. T cell exhaustion is an anthropomorphic term used to indicate the programmed response of immune cells that occurs in the setting of persistent antigen exposure and T cell receptor (TCR) stimulation [24] and leads to progressive curtailing of T cell polyfunctionality. While per se not necessarily a dysfunctional state—indeed it appears evolutionarily conserved to protect tissues from excessive immune-mediated injury—T cell exhaustion reduces the ability to recognize and eradicate infected or neoplastic cells. Exhausted T cells are identified functionally and by persistently high expression of multiple inhibitory receptors, including cytotoxic T lymphocyte antigen 5 (CTLA-4), programmed cell death protein 1 (PD-1), T cell immunoglobulin and mucin domain 3 (TIM3), lymphocyte activation gene 3 (LAG3), and T cell immunoreceptor with Ig and ITIM domains (TIGIT) [25]. CTLA-4, PD-1 and its ligand PDL-1 have been developed as druggable targets, whereby their blockade re-establishes T cell activity. Importantly, it appears clear from animal models and human disease that the pool of exhausted T cells is phenotypically and functionally heterogeneous [26], with only a fraction of cells, most notably represented by TCF1 + memory-like T cells, able to respond to checkpoint blockade [27, 28, 29••, 30]. The proportion of these T cell subpopulations, versus terminally differentiated exhausted cells incapable of rescue, may greatly influence overall treatment response [31]. The tumor microenvironment itself has been identified as an important factor in treatment response, with PDL1 expression in biopsy samples already an FDA-approved predictive biomarker and the number and spatial arrangement of tumor infiltrating-cells appearing promising [20, 21].

The shared prominence of T cell exhaustion between cancer and HIV [32], as well as the success of ICIs for some cancers [17], have provided fuel for the exploration of ICIs for treating not only cancers but also opportunistic infections in PLWH.

## Use of Checkpoint Inhibitors in HIV Populations

The advancement of antiretroviral therapy (ART) for PLWH has significantly reduced the risk of dying from AIDS-related illnesses. Despite this, PLWH still today have an increased risk of developing and dying from both AIDS-defining and non-AIDS-defining cancers [33, 34]. However, PLWH have been largely excluded from clinical trials investigating ICI for cancer treatment [35], due to concerns regarding the risks of activating an immune response against HIV-infected host cells, as well as the concern of developing immune-related adverse events (irAEs) across organ systems [32]. Recognizing the increased risk of incidence and mortality from cancers in populations with HIV [33, 34], as well as the benefit of ICIs for some cancers [17], more recent studies have sought to include PLWH in ICI clinical trials.

The application of ICIs for cancer treatment in a PLWH was first reported in a 2011 case report, with the use of ipilimumab for treating metastatic melanoma [36]. The therapy was tolerated well, showing reduced size of all four liver lesions and no adverse effect on HIV viral load or CD4 + cell counts. ICIs have since been applied in other PLWH. Kim and Cook [37] published a systematic review in 2019, detailing 73 PLWH who received ICI therapy for advanced-stage cancer. Of 28 patients with undetectable HIV load and paired pre-treatment and post-treatment data, 26 patients (93%) continued to have HIV levels below the limit of detection and essentially stable CD4 cell counts (mean [SD] increase, 12.3 [28.5]/μL). Six patients (8.6%) reported grade 3 or higher irAEs, further suggesting ICI therapy was generally well tolerated. Notably, there was no significant difference in efficacy of ICI therapy between patients with and without HIV, with response rates of 63% for Kaposi sarcoma, 30% for non-small cell lung cancer, and 27% for melanoma [37]. ICIs continue to be explored in PLWH, with recent studies supporting the safety of pembrolizumab for people taking ART [38••], as well as in combination with chemotherapy [39]. These findings suggest that ICI may be a safe and efficacious therapy for PLWH.

Recognizing the accumulating evidence supporting the safety and efficacy of ICI therapy in patients with HIV, the FDA published a guidance statement advocating the inclusion of people with HIV in ICI trials [40]. Despite this, a recent study by Vora et al. in 2021 concluded that the population with HIV continues to be excluded from most ICI trials [35]. Including PLWH in ICI clinical trials is necessary to provide access to groundbreaking treatments for this vulnerable population.

## Effect of Checkpoint Inhibitors on Markers of HIV Infection

The HIV reservoir, comprised of latently infected host cells carrying the integrated viral genome, presents challenge for achieving permanent viral suppression in PLWH [41]. CD4 + cells with increased expression of immune checkpoints, such as PD-1, are major sources of replication-competent virus [42], and their levels correlate with harbored HIV DNA levels [43, 44]. This suggests that ICIs may impact HIV replication by potentially targeting latently infected cells.

In vitro, an ICI blocking PD-1 engagement increased the survival and proliferation of HIV-specific CD8 + T cells, as well as enhanced cytokine production to antigen exposure [45]. In vitro studies targeting immune checkpoints suggest an effect on reversing HIV latency [46, 47]. The impact of ICI on the HIV reservoir was described for the first time in a patient with HIV infection with metastatic melanoma in 2015 [48]. In addition to a decrease in plasma HIV RNA, there was a significant difference in number and phenotype of CD4 + T cells, predominantly an increase in total memory and effector memory CD4 + T cells [48].

In 2018, a case report by Guihot et al. [49••] described a profound decrease in the HIV reservoir following nivolumab treatment in an HIV-infected patient via a “shock and kill” mechanism—a brief reactivation of HIV replication within CD4 + T cells and a reduction in exhausted CD4 + and CD8 + T cells, followed by a dramatic increase in HIV-specific CD8 + T cells [49••]. However, a subsequent 2018 study detailed 3 HIV-positive patients receiving either nivolumab or pembrolizumab for cancer and with no consistent effect on HIV-specific T cell responses [50]. These inconsistent findings prompted a thorough systematic review in 2020 of the impact of ICI on the HIV reservoir [51]. Among 176 participants included, HIV plasma loads remained unchanged in 91.9%, and CD4 + cell count remained stable in 60.7%, increased in 24.6%, and decreased in 14.7%. More recently, a prospective study in 32 people with HIV on ART and cancer found that treatment with pembrolizumab led to modest transient increase in median viral transcription [52], while a separate study in 32 PLWH and cancer found a decrease in cell-associated HIV-DNA in 6 patients while 4 had transient increase [53]. Taken together, these findings suggest the potential of ICI for reversing viral latency is likely limited and variable, but worthy of further investigation. While PD-1 is effectively blocked on T cells in the cerebrospinal fluid (CSF) compartment following systemic administration [54••], to what extent drug reaches HIV reservoir-harboring cells in the brain parenchyma is unknown. An ongoing study investigating CNS effects of pembrolizumab for patients with HIV on ART (NCT03239899) addresses this open question.

These studies do however clearly demonstrate that ICI therapy is generally well tolerated among HIV populations. This, combined with ICI’s success in treating cancers and other chronic infections in PLWH, has opened the door for their investigation in HIV-associated PML.

## Safety and Efficacy of Checkpoint Inhibition for PML in HIV

The involvement of the PD-1 pathway in PML contributes to the rationale for exploring ICIs to restore the immune response and improve viral clearance. Patients with PML display higher percentages of PD-1^+^ CD4^+^ and CD8^+^ T cells in the CSF and blood [54••, 55] and increased expression of PD-1 on JCV-specific CD8^+^ cytotoxic T-lymphocytes [55]. Moreover, blocking PD-1 in vitro resulted in an increase in IFN-γ expression in JCV-stimulated CD8^+^ T cells in an HIV-positive patient with active PML [55] and increased PD-1 and PD-L1 expression has been described in post-mortem PML lesion tissue [54••]. These findings suggest that targeting the PD-1 pathway through checkpoint inhibition may be an effective strategy to improve the cellular immune response to JCV.

ICIs have been explored in limited cases of patients with PML for whom immune reconstitution was not readily achieved and thus had low expectation for survival. As of April 15, 2022, only 5 of the 53 published cases of treated PML [5, 54••, 56–67, 68••, 69–74, 75••, 76–80, 81••, 82], although the number of unpublished cases of treated PML, both related to HIV and otherwise, exceeds this (personal communication). Nevertheless, evaluating the overall available experience and outcome of checkpoint inhibition in PML will likely provide helpful insights when translating this therapy’s potential to the HIV-PML population (Supplemental Table). Of the published patients, 41 (77%) received pembrolizumab, 11 (21%) received nivolumab, and a single case was treated with atezolizumab. In some cases, checkpoint inhibition demonstrated favorable outcome, with enhanced JCV-specific CD4 + and CD8 + T cell immune response [54••, 78, 82], a decline in JCV CSF copy number [54••, 56, 57, 62, 72, 73, 75••, 79, 83], and stable or improved clinical course [54••, 62, 67, 68••, 72–74, 75••, 78–80, 81••, 82].

Published cases of HIV-related PML treated with ICI are summarized in Table 1. Of the five patients with HIV-related PML, four were treated with pembrolizumab [54••, 75••, 81••]. Of these, one was newly stable on ART and two had a long history of ART, both with plasma HIV viral load less than the limit of detection at the time of pembrolizumab treatment; the fourth had a history of ART non-adherence and restarted ART contemporaneously to ICI treatment due to rapid worsening of PML. Of note, this last patient had evident contrast enhancement on baseline MRI [81••]. Three had pre- and post-treatment JCV CSF viral load measurements and exhibited a decrease between baseline and final visit upon treatment with ICI [54••, 75••]. Three showed significant improvement of neurological deficits and decrease in lesion size by MRI, with no recurrence of PML at last follow-up [54••, 75••, 81••]. One patient experienced clinical and radiological stabilization associated with increase in JCV-specific T cell response within 1 month of the first dose of pembrolizumab. This patient received an additional dose of pembrolizumab a year later due to renewed worsening of clinical symptoms and imaging, again followed by decline in CSF JCV load [54••]. None of the four developed notable adverse events nor PML-IRIS [54••, 75••, 81••].

**Table 1:** Summary of published cases of HIV-PML treated with ICI (as of April 15, 2022)

Age/sex (reference)	Time since HIV diagnosis	Time since ART initiation	Time since PML diagnosis	Checkpoint inhibitor, dose, interval, number of doses	CD4/CD8/CD19 counts baseline (cells/mm^3^)	CD4/CD8/CD19 counts after ICI (cells/mm^3^)	JCV in CSF baseline and final value (copies/mL)	Plasma HIV RNA baseline and final (copies/ml)	IRIS?	Outcome	Adverse events
48/female [54••]	~ 20 years	7 months	7 months	Pembrolizumab 2 mg/kg Q4 weeks (× 3)	117/933/553	158/633/710	63 and undetected	< 20 and < 20	No	Significant improvement in naming, language fluency, and cognition Decreased in modified Rankin score Progressive decreased in PML lesion size on MRI	No
58/male [54••]	~ 18 years	~ 18 years	3 weeks	Pembrolizumab 2 mg/kg Q4 weeks (× 2)	580/1588/200	524/1584/168	286 and 98	< 20 and < 20	No	Subjective decline of symptoms at month 11, then subjective symptom improvement after the additional dose	No
53/male [68••]	Unknown	Unknown	Unknown	Nivolumab 3 mg/kg (× 1)	164/474/14	Unknown/unknown/unknown	457 and unknown	Unknown and unknown	No	Rapid clinical and radiologic worsening Died of PML	unknown
44/female [75••]	~ 19 years	~ 19 years	Recent, time not specific	Pembrolizumab 2 mg/kg (× 4)	120/unknown/unknown	150/unknown/unknown	Positive and negative	< 20 and < 20	No	Gradual improvement of ataxia, strength and dysarthria associated with radiological improvement by 3 months	No
43/male [81••]	~ 10 years	Contemporaneous to ICI	weeks	Pembrolizumab 2 mg/kg (× 2)	20/unknown/unknown	20/unknown/unknown	Undetected (diagnosis made by brain biopsy)	9.9 × 10^5^ and unknown	?contrast enhancement noted on presenting scan; no radiological or clinical evidence of IRIS following ICI	Neurological symptoms improved within 1 month. Patient lost to follow-up × 2 years, but reengaged care 2 years later. At follow-up he was neurologically stable although non-adherence with HIV with CD4 count of 60 and HIV viral load of 6.5 × 10^5^ copies/ml	No

Roos-Weil et al. [68••] documented a published case using nivolumab for HIV-associated PML in an individual with concomitant T cell lymphoma. This patient presented with severe disability (modified Rankin score of 5) and a JCV CSF viral load of over 200,000 copies/mL at the time of treatment. Unlike the previous report, this patient rapidly worsened following the first infusion, with no evidence of PML-IRIS by MRI, and death was attributed to progression of PML shortly thereafter [68••].

An optimal response to checkpoint inhibitors can be conceptualized as a balance between antigen burden and appropriate degree of immune reinvigoration. Suboptimal outcomes could therefore result on one end from inadequate reinvigoration, or on the other, from excessive inflammatory response leading to irAEs or, in the case of PML, to PML-IRIS (Fig. 1). At least two of the reported cases of HIV-related PML treated with ICI, the modest CSF viral load and disability (modified Rankin score of 3 and 2) at presentation [54••], suggesting relatively less advanced disease and possibly less severe immune exhaustion, may have favored their positive outcome compared to the case with poor outcome [68••].

Evaluating the collective published experience of ICI for the treatment of non-HIV related PML may provide further insight into what other factors are important to consider when weighing the likelihood of response to checkpoint inhibition. High CSF JCV load and severe disability suggesting advanced or aggressive PML, as well as severe lymphopenia, intrinsic T cell dysfunction, or ongoing treatment with immune suppressive therapies, each conceivably associated with decreased ability to mount any immune response, appear to be common features among cases with poor outcome [54••, 59, 61, 76, 79, 80, 84]. The presence of detectable JCV-specific T cells at baseline may on the other hand be a positive indicator [54••, 61, 78, 82], with similar observations seen in predicting ICI response in cancer [85]. This is further supported by in vitro evidence that PD-1 blockade resulted in an increase in JCV-specific IFN-γ immune response in healthy volunteers, but only in those with a detectable number of JCV-specific CD8 + T cells at baseline [39].

## Challenges of Checkpoint Inhibitor Therapy for PML in HIV

In our center, HIV-related PML is most commonly observed in untreated HIV infection but can also occur in the setting of ART non-adherence or resistance. Standard-of-care for ART-naive patients with PML is first and foremost represented by immediate initiation of ART, which alone is associated with survival of more than half of cases. For patients with suboptimal HIV suppression related to non-adherence or resistance, first-line treatment is to optimize the ART regimen; with current potent ART regimens, there is no clear evidence suggesting improved outcome of PML using therapies with higher CNS penetration effectiveness score (CPE) [86–88]. The appropriate timing to consider adjunctive strategies to augment JCV anti-viral responses beyond standard-of-care is not established. Adjunctive strategies likely would be reserved for cases with low likelihood of achieving timely immune reconstitution in the face of progressing disease. While checkpoint inhibition may represent one possible consideration, several risks must be weighed.

The main concern is the risk of developing immune reconstitution inflammatory syndrome (IRIS). IRIS is a dysregulated, hyper-inflammatory response that can result in immune-mediated damage. In HIV-associated PML, IRIS is characterized by a worsening of neurological symptoms associated with an increase in CD4 + T cell count and a reduction in HIV RNA plasma levels [1]. The inflammatory features of PML-IRIS can be appreciated by MRI, with contrast enhancement, edema, and/or mass effect [89]. IRIS can be distinguished into “paradoxical IRIS,” occurring when symptoms of a known PML diagnosis worsen and “unmasking IRIS” when the inflammatory response to a previously inapparent infection results in new clinical symptoms [1]. While re-invigorating the immune system is an effective strategy to improve viral clearance, introducing checkpoint inhibition in combination with ART could enhance the risk of developing IRIS. Indeed, studies suggest as many as 25–30% of HIV patients on ART develop IRIS [90], and, specifically in the setting of PML, an estimated 28% of PML-IRIS cases are fatal [91]. Adjunctive therapy with checkpoint inhibition in patients newly initiating or escalating ART could compound the risk of developing IRIS. Importantly, the window for developing symptomatic IRIS is broad and unpredictable, occurring anytime up to 26 weeks after ART initiation [92]. This highlights a potentially prolonged continued risk for triggering IRIS in scenarios where ART does not quickly achieve adequate immune reconstitution to control infection and ICIs are considered.

The specific risk for developing PML-IRIS following ICI is not known. The majority of reported cases that used ICI as an experimental strategy for PML included patients with profound immune suppression that could not otherwise be reversed, and so the relatively low occurrence of IRIS in this setting is likely not generalizable to the HIV population. Independently of ICIs, known risk factors for developing IRIS include a history of multiple opportunistic infections, low starting CD4 count, and rapid decrease in HIV plasma viral load following initiation of ART [92, 93]. Close monitoring for development of PML-IRIS and timely management thus become particularly critical in the context of checkpoint inhibition. Steroids are the mainstay of treatment for PML-IRIS, although there is no standardized dosing schema. It is important to consider that corticosteroids dampens the JCV-specific cellular immune response, and while they have established benefit for the treatment of clinically manifest PML-IRIS, their use for prevention of IRIS is not recommended [94, 95].

Another challenge of checkpoint inhibition to note is the concern of triggering irAEs. irAEs have been reported across organ systems in response to ICIs, and can lead to colitis, pneumonitis, rash, or other organ dysfunctions [32]. Most irAEs in response to ICI are mild, but some can be serious and even life-threatening [96].

In two reported cases, PML has been diagnosed following checkpoint inhibition for other infections. One reports the use of nivolumab for a PML patient with stage IV Hodgkin lymphoma in 2018. To note, the patient’s lymphoma and steroid use rendered the patient highly immunocompromised. Therefore, it is difficult to conclude if the development of PML was due to the nivolumab, or rather an unfortunate complication of multiple factors [97]. In the second case report in 2019, a patient with stage IV Hodgkin lymphoma given nivolumab then displayed clinical signs of PML and IRIS diagnosed by brain biopsy, consistent with unmasking PML [58]. This patient survived 3 years, which significantly exceeds the 2-month median survival for PML patients with hematological malignancy [98]. Checkpoint inhibition thus appears to have promoted effective immune reconstitution to control the unmasked PML. As checkpoint inhibitors become more broadly applied to immunocompromised patients, the incidence of unmasking PML may increase [4].

## Conclusions

PML is a devastating disease that continues to pose a significant burden due the lack of JC virus-specific treatment options. Still today, HIV accounts for a significant proportion of PML cases [11••].

ICIs have garnered much attention due to their success in treating numerous cancers. Their mode of action, represented by re-invigoration of the immune system, could be beneficial for promoting antiviral responses in PML, and more specifically in HIV-related PML when ART fails to control infection. Although PLWH have largely been excluded from ICI clinical trials [24], accumulating evidence demonstrates that ICIs are generally well tolerated in populations with HIV infection [26].

Studies exploring ICIs for patients with PML thus far have shown heterogenous results, not dissimilar to the experience in oncology. Nevertheless, ICIs hold promise as a strategy to promote immune reconstitution in selected cases of PML, and possibly HIV-associated PML. The specific risk of ICI in promoting PML-IRIS or IRIS in general is not completely understood, nor how this risk might affect appropriate timing of treatment or patient selection.

Robust biomarkers predictive of ICI treatment response are lacking. The experience available to date suggests profound lymphopenia and absence of any detectable anti-JCV activity are associated with poor likelihood of treatment response. Furthermore, study of ICI-responsive T cell subpopulations may provide insight into the inconsistencies observed, as well as into strategies to improve efficacy across indications. Formal, prospective investigations of ICI are necessary to determine whether ICI should be included in treatment escalation regimens of HIV-related PML.

## Supplementary Information

Below is the link to the electronic supplementary material.

Supplementary file1 (DOCX 40 KB)
