# Supplementary material for: Promise and Challenges of Checkpoint Inhibitor Therapy for Progressive Multifocal Leukoencephalopathy in HIV
Source: Curr HIV/AIDS Rep. 2022 Oct 1;19(6):580–91. doi: 10.1007/s11904-022-00626-w (PMC9759507; doi:10.1007/s11904-022-00626-w)
Supplement: Supplementary file 1 — Supplementary file1 (DOCX 40 KB) [file 11904_2022_626_MOESM1_ESM.docx]

**Supplemental Table:** Summary of published cases of checkpoint inhibitors for treatment of PML as of April 15, 2022

| **PMID** | **First author, Year** | **Age/sex** | **Underlying Disease Diagnosis** | **Checkpoint inhibitor, dose, interval, number of doses** | **CD4 / CD8 / CD19 counts baseline (cells/mm^3^)** | **JCV in CSF baseline and final value (copies/mL)** | **IRIS?** | **Outcome** | **Adverse Events** |
| --- | --- | --- | --- | --- | --- | --- | --- | --- | --- |
| 30969503 | Cortese et al. 2019 | 48/ female | *HIV | Pembrolizumab  2 mg/kg body weight  Q4 weeks (x3) | 117 / 933 / 553 | 63 and undetected | No | Clinical and MRI improvement | No |
|  |  | 58/  male | *HIV | Pembrolizumab  2 mg/kg  Q4 weeks (x2) | 580 / 1588 / 200 | 286 and 98 | No | Slight clinical improvement, MRI unchanged | No |
|  |  | 67/  male | Chronic lymphocytic leukemia | Pembrolizumab  2 mg/kg  Q4 weeks (x3) | 136 / 194 / 406 | 232 and 176 | No | Stabilized before injection; no benefit from ICI  Survived PML | No |
|  |  | 78/  male | Chronic lymphocytic leukemia | Pembrolizumab  2 mg/kg  Q4 weeks (x2) | 94 / 104 / 10 | 6,044 and 338 | No | Decline in JC viral load  Survived PML  Died of underlying cancer | No |
|  |  | 69/ female | Non-Hodgkin lymphoma | Pembrolizumab  2 mg/kg  (x1) | 158 / 47 / 26 | 26,494 and 22,948 | No | Continued decline of neurologic symptoms  Died of PML within six months | No |
|  |  | 31/  male | Idiopathic lymphopenia | Pembrolizumab  2 mg/kg  Q4 weeks (x3) | 357 / 68 / 60 | 5,248 and undetected | Yes | Clinical improvement  Survived PML | Worsening of psoriatic rash |
|  |  | 62/ female | Idiopathic lymphopenia | Pembrolizumab  2 mg/kg  Q4 weeks (x2) | 13 / 330 / 339 | 28,350 and 23,194 | No | Continued decline of neurologic symptoms  Died of PML within 6 months | Maculopapular rash |
|  |  | 70/  male | Hodgkin’s lymphoma | Pembrolizumab  2 mg/kg  (x1) | 127 / 199 / 103 | 261 and undetected | No | Survived PML  Died of underlying cancer | No |
| **30969500** | Walter et al. 2019 | 60/ female | Idiopathic lymphopenia | Nivolumab  240 mg  Q2 weeks (x8) | 8 / 42 / 5 | 200,000 and 200 | Yes | Stabilization of neurologic symptoms  Survived PML | No |
| **30864100** | Hoang et al. 2019 | 65/ female | Hodgkin’s lymphoma with history of diffuse large B cell lymphoma | Nivolumab  3 mg/ kg  Q2 weeks (x8) | unknown / unknown / unknown | unknown and unknown | Yes | Clinical signs of PML after injection, diagnosed by brain biopsy (unmasking PML)  Survived PML  Died of underlying lymphoma | No |
| **31625858** | Medrano et al. 2019 | 81/  male | Kidney transplant | Nivolumab  3 mg/ kg  Q15 days | 76 / 56 / unknown | 3162 and unknown | No | Died of PML within 8 weeks of diagnosis | No |
|  |  | 77/  male | Kidney transplant | Nivolumab  3 mg/ kg  Q15 days | 162 / 106 / unknown | 794 and unknown | No | Died of PML within 8 weeks of diagnosis | No |
|  |  | 67/ female | Kidney transplant | Nivolumab  3 mg/ kg  Q15 days | 287 / 67 / unknown | 794 and unknown | No | Died of PML within 8 weeks of diagnosis | No |
| **31597693** | Küpper et al. 2019 | 42/  male | Primary immunodeficiency syndrome | Pembrolizumab  2 mg/kg  Q2 weeks (x5) | 150-180 / unknown / unknown | 38 and unknown | No | Died of PML within 6 months of diagnosis | No |
| **31597692** | Pawlitzki et al. 2019 | 38/  male | IgG and IgA deficiency and Behçet disease | Pembrolizumab  2mg/kg  Q3 weeks (x2) | 181 / 511 / unknown | 2,561,955 and 7,685,000 | No | Clinical worsening  Died of PML | No |
| **31660340** | Audemard-Verger et al. 2019 | 53/  male | Unknown (history of silicosis) | Nivolumab  1.5 mg/ kg  Q2 weeks (~x20) | 365 / 616 / 113 | ~1,995 and undetectable | No | Stabilization of clinical symptoms after first dose, then improvement by 6 months | No |
| **32910430** | Holmes et al. 2020 | 61/ female | Large B cell lymphoma | Pembrolizumab  2 mg/kg  Q1 month (x3) | 304 / 340 / unknown | (Positive but value not stated) and undetected | No | Clinical stabilization  Minimal MRI improvement | unknown |
| 32434801 | Mahler et al. 2020 | 33/ female | Large B cell lymphoma | IL-2 therapy followed by  Pembrolizumab  200 mg  (x1) | 24 / 4 / unknown | 449 and 98 | No | Clinical stabilization  CD4 and CD8 increased  Lymphoma reccurred | No |
|  |  | 60/  male | Large B cell lymphoma | IL-2 therapy followed by  Pembrolizumab  175 mg  (x1) | 245 / 192 / unknown | 309 and undetected | No | CD4 and CD8 increased  Stabilization of PML lesions  Clinical improvement to full independence of all activities of daily living | No |
| **32644211** | Goereci et al. 2020 | 71/  male | Unknown | IL-2 therapy followed by  Pembrolizumab  2 mg/kg  Q1 month (x3) | unknown / unknown / unknown | 1000 and undetected | No | Clinical and radiological improvement, followed by recurring respiratory distress and death at 6 weeks following pembrolizumab treatment | Possible bronchopulmonary symptoms |
| **32644207** | Grassl et al. 2020 | 69/  male | Sezary syndrome | Nivolumab  240 mg  Q2 weeks (x3) | 1180 / 238 / unknown | 4505 and "slightly increased" | No | Clinical worsening  Progression in PML lesion size and number  Died of PML within 5 weeks | No |
| **32700225** | Stogbauer et al. 2020 | 54 /female | Large B cell lymphoma | Pembrolizumab  2 mg/kg  Q4 weeks (x3) | unknown / unknown / unknown | 3,500,000 and unknown | Yes | Clinical and MRI worsening, Died of PML | No |
| **32549952** | Kapadia and Ney, 2020 | 69/  female | Large B cell lymphoma | Pembrolizumab  Unknown  (x4) | Unknown/  unknown/  unknown | Unknown (diagnosed by brain biopsy) | No | Clinical improvement by month 4 with MRI stabilization  Survived PML | No |
| **32005559** | Mozo Ruiz et al, 2020 | 44/  female | HIV | Pembrolizumab  2mg/kg  Q4 weeks (x3) | 120/  unkown/  unknown | Positive and undetected | No | Clinical and radiological improvement  Survived PML | No |
| **31797364/33515299** | Uzunov et al. 2020/ Roos-Weil 2021 | 47/ female | Acute myeloid leukemia | Nivolumab  1 mg/kg (x2)  3 mg/kg (x1)  (Q2 weeks) | unknown / unknown / unknown | 47,377 and undetectable | No | Clinical improvement within 1 week  Reduced PML lesions on MRI  CSF JCV undetectable after 4 weeks  Survived PML | Myositis |
| **33515299** | Roos-Weil et al. 2021 | 73/  male | Large B cell lymphoma | Nivolumab  3 mg/kg  Q2 weeks (x3) | 53 / 116 / unknown | Undetected (brain biopsy diagnosis) | No | Clinical stabilization, MRI atrophy | No |
|  |  | 53/  male | *HIV  T- cell lymphoma | Nivolumab  3 mg/kg  (x1) | 164 / 474 / unknown | 457 and unknown | No | Rapid clinical and MRI worsening  Died of PML | unknown |
|  |  | 75/  male | Follicular lymphoma | Pembrolizumab  2 mg/kg  (x1) | 106 / 58 / unknown | 125,719 and unknown | No | Rapid clinical and MRI worsening  Died of PML | No |
|  |  | 71/  male | Myasthenia gravis | Pembrolizumab  2 mg/kg  Q4 weeks (x4) | 152 / 333 / unknown | 110,686 and unknown (stable, precise value unknown) | No | Clinical worsening following by clinical and MRI stabilization | No |
|  |  | 42/ female | CVID  Digestive tract granulomatosis | Nivolumab  3 mg/kg  Q2 months (x2) | 305 / 89 / unknown | 329 and unknown (stable, precise value unknown) | No | Clinical improvement within 4 weeks, injections stopped due to adverse event, led to rapid PML progression and death | Colitis / hepatitis |
| **34039733** | Beudel et al. 2021 | 73/  male | Idiopathic lymphoma | Pembrolizumab  200 mg  (x1) | 190 / 90 / unknown | 20,000 and unknown | No | Clinical worsening for 3 weeks followed by clinical improvement and MRI stabilization | No |
|  |  | 58/ female | Polymyositis and systemic sclerosis | Pembrolizumab  200 mg  (x1) | 170 / 70 / unknown | unknown and unknown | No | Clinical and MRI improvement | No |
| **34035834** | Möhn et al. 2021 | 78/  male | Mantle cell lymphoma | Pembrolizumab  2 mg/kg  Q4 weeks (x4) | unknown / unknown / unknown | <500 and 220 | Possible (CE on MRI) | Reduced JC viral load, clinical and MRI worsening | No |
|  |  | 73/  male | Immunocytoma | Pembrolizumab  2 mg/kg  Q4 weeks (x3) | unknown / unknown / unknown | Undetected and unknown | No | Clinical and MRI improvement | No |
|  |  | 70/ female | Follicular lymphoma | Pembrolizumab  2 mg/kg  Q? (x2) | unknown / unknown / unknown | 90,000 and 190,000 | Possible (CE on MRI) | Clinical and MRI worsening  Died of PML within 3 weeks of second dose | No |
| **33724354** | Patel et al, 2021 | 78/  female | Multiple sclerosis treated with ocrelizumab | Pemrolizumab  200mg | 294/85/1 | 1000 and unknown | No | Rapid deterioration with no signs of IRIS  Died of PML | No |
| **34484921** | Haroche et al, 2021 | 70/  female | Erdheim-Chester Disease | Nivolumab  Unknown  (x2) | 426/  unknown/  unknown | 250 and unknown | Yes | Clinical deterioration following 2^nd^ dose  Died of pneuomonia | No |
| **34285093** | Wicklein et al, 2021 | 57/  female | Marginal zone lymphoma | Pembrolizumab  2mg/kg  Q2-6 weeks (x5) followed by BKV-specfiic T cells x2 followed by pembrolizumab 2mg/kg x 5 | Unknown/  unknown/  unknown | 40 and 594 (peaked at 10,000) | No | Initial clinical worsening followed by MRI stabilization and some clinical improvement | No |
| **34051624** | Fischbach et al. 2021 | 65/  male | Idiopathic CD4+ lymphocytopenia | Nivolumab  240 mg  Q2 weeks (x23) | unknown / unknown / unknown | 1024 and unknown | No | Initial clinical worsening followed by MRI stabilization and slight clinical improvement | Mucositis / bronchopulmonary infection |
| **34325541** | Lan et al. 2021 | 62/ female | Systemic lupus erythematosus | Pembrolizumab  2 mg/kg  Q4 weeks (x6) | unknown / unknown / unknown | 2,300,000 and 58,000 | No | Clinical stabilization then clinical and MRI improvement later  Died of pneumonia | No |
| **34196768** | Volk et al. 2021 | 21/  male | CD40-ligand deficiency | Pembrolizumab  2 mg/kg  Q5 weeks (x2)  Q8 weeks (x1) | 457 / 1577 / 479 | 471 and undetected | Possible (CE on MRI) | Initial clinical worsening followed by clinical improvement and MRI stabilization | Maculopapular rash |
|  |  | 45/ female | CVID with autoimmune cytopenia and interstitial lung disease | Pembrolizumab  2 mg/kg  Q3 weeks (x2)  Q4 months (x1)  0.5 mg/kg  (x1) | 142 / 37 / 0 | Undetected (PCR from brain biopsy was positive) and unknown | Possible (CE on MRI) | Clinical worsening  Developed several adverse events requiring corticoid therapy and granulocyte colony-stimulating factor  Died possibly due to autoimmune conditions | Diarrhea / severe pancytopenia |
|  |  | 78/  male | Diffuse large B-cell lymphoma (DLBCL) | Pembrolizumab  2 mg/kg  Q25 days (x2) | 500 / 265 / 0 | <500 and undetected | No | Lymphoma relapsed  Stable PML symptoms  Died of intracranial hemorrhage | No |
|  |  | 45/  male | CID due to DOCK8 deficiency, history of meningitis | Pembrolizumab  2 mg/kg  Q20 days (x2)  Q30 days (x1) | 261 / 282 / 195 | 500 and 68,500 | No | Rapid clinical worsening  Died of PML | Maculopapular rash |
|  | Rauer et al. 2019/ Volk et al. 2021 (previously published) | 49/  male | CVID, DLBCL | Pembrolizumab  2 mg/kg  Q2-4 weeks  Total 24 infusions | 40 / 62 / 0 | 119,000 and undetectable | Yes | Initial clinical improvement then stabilization  Survived PML | Diarrhea / transaminitis |
| **34586433** | Nitsch et al, 2021 | 60/  female | Follicular lymphoma | Pembrolizumab  2mg/kg  Q4 weeks (x5) | Unknown/  Unknown/  0 | Positive and undetected | Yes | Clinical and radiological improvement  Survived PML | No |
|  |  | 76/  female | Marginal zone lymphoma | Pembrolizumab  2mg/kg  Q4 weeks (x5) | Unknown/  Unknown/  0 | Undetected (diagnosed by brain biopsy) | Yes | Clinical and radiological improvement  Survived PML | No |
|  |  | 53/  female | Pulmonary adenocarcinoma | Pembrolizumab  2mg/kg  Q3 weeks | Unkown/  Unknown/  unkown | 570,000 and  undetected | Yes | JCV in CSF declined to undetected by second infusion; clinical worsening with evidence of IRIS by MRI; treated with corticosteroids but course complicated by pneumonia and seizures.  Patient died of complications of IRIS | No |
| **34874539** | Darcy et al, 2021 | 60/  male | Rheumatoid arthritis | Pembrolizumab  Unknown  Q3 weeks (x2) | Unknown/  unknown/  unknown | Log 3.11 | No | Continued clinical progression, followed by stabilization and survival | No |
|  |  | 77/  male | Chronic lymphocytic leukemia | Pembrolizumab  2mg/kg  Q3 weeks (x2) | Unknown/  unknown/  unknown | Unknown (diagnosed by brain biopsy) | No | Continued clinical progression  Died of PML | No |
| **34856110** | Lambert et al, 2022 | 77/  female | Chronic lymphocytic leukemia | Atezolizumab  1200mg  Q3 weeks (x | 280/  80/  30 | 733,845 and 945 | Yes | Clinical and virological improvement; IRIS treated with corticosteroids, associated with subsequent progression of PML and rise in CSF JCV copy number  Patient died of complications of PML (aspiration pneumonia) | Psoriasis-like skin lesions, abdominal pain, transient 3^rd^ degree atrioventricular block |
| **35320511** | Boesl et al, 2022 | 69/  unknown | Follicular lymphoma | IVIg 2g/kg x 5 days followed by pembrolizumab 2mg/kg  Q3-4 weeks (x7) | 214/  unknown/  unknown | Positive | No | Early clinical and later radiological improvement  Survived PML | No |
| **35084387** | Sim et al, 2022 | 43/  male | HIV | Pembrolizumab  2mg/kg  Q4 weeks (x2) | 20 x 10^6^/L  Unknown/  unknown | Undetected (diagnosed by brain biopsy) | ? | Clinical and radiological improvement  Survived PML | No |

CVID=Common variable immunodeficiency; CE=Contrast-enhancement; ICI=Immune checkpoint inhibitor
